# Isolated from Populus euphratica rhizosphere soil, and mining their metabolites

**DOI:** 10.3389/fmicb.2025.1530786

**Published:** 2025-02-19

**Authors:** Jia-xin Zhang, Yi-huang Chen, Xinrong Luo, Li-li Zhang, Xiao-xia Luo

**Affiliations:** ^1^Key Laboratory of Protection and Utilization of Biological Resources in Tarim Basin of Xinjiang Production & Construction Corps, Xinjiang, China; ^2^College of Life Sciences and Technology, Tarim University, Xinjiang, China

**Keywords:** Taklimakan Desert, *Streptomyces solitudinis* sp. nov, genome analysis, *Streptomyces rubellus* sp. nov, aminoglycosides

## Abstract

**Introduction:**

The microbial community in desert ecosystems is a vital and highly active component. *Streptomyces*, one of the dominant genera within this community, exhibits significant capabilities in metabolic degradation and the synthesis of secondary metabolites.

**Methods:**

To investigate the metabolic potential of *Streptomyces desertis*, two strains of *Streptomyces* were isolated from rhizosphere soil samples of *Populus euphratica* in the Taklimakan Desert during the initial phase of this study, TRM 70351^T^ and TRM 70361^T^.

**Results:**

The identification of these strains as belonging to the genus *Streptomyces* was confirmed through 16S rRNA sequencing. All calculated Average Nucleotide Identity (ANI) values were below the 95% cut-off recommended for distinguishing distinct species, and the estimated digital DNA-DNA hybridization (dDDH) values were all below the suggested threshold of 70% for species delineation. Results from phylogenetic, genomic, phenotypic, and chemotaxonomic analyses confirmed that TRM 70351^T^ and TRM 70361^T^ represent two new species within the genus *Streptomyces*, designated as *Streptomyces solitudini*s sp. nov. and *Streptomyces rubellus* sp. nov. The type strain for *Streptomyces solitudinis* sp. nov. is TRM 70351^T^ ( = CCTCC AA 2020049^T^ = LMG 32404^T^), while the type strain for *Streptomyces rubellus* sp. nov. is TRM 70361^T^ ( = CCTCC AA 2020043^T^ = JCM 35793^T^). Notably, Among the identified gene clusters of TRM 70351^T^, cluster 12.1 was predicted to be the biosynthetic gene cluster responsible for producing the aminoglycoside compound streptomycin, exhibiting a similarity of 55%. In this study, HSQC-TOCSY was employed to detect the presence of aminoglycosides in fermentation medium No. 1, while LC-MS/MS was utilized to analyze the molecular fragments of neomycin and streptomycin in the alkaline aqueous phase sample of the fermentation product. The mixture was eluted using methanol and ammonia water in a 3:1 ratio, leading to the further separation of the compounds daidzein and Tridec-1-ene.

**Discussion:**

This study has enhanced the species resources of *Streptomyces deserticum* and the diversity of aminoglycoside compound-producing bacteria. TRM 70351^T^ exhibited unique metabolic potential, indicating that further studies could be conducted in the future.

## Introduction

Few plants and animals can survive in deserts, particularly in the desert hinterland, making the microbial community the most active and crucial component of this ecosystem. *Actinomycetes*, characterized by their strong spore-producing capabilities, metabolic degradation abilities, synthesis of secondary metabolites, and various ultraviolet repair mechanisms, are among the dominant phyla in desert environments, with *Streptomyces* being one of the predominant genera (Ensign, 1978; Gao and Garcia-Pichel, 2011; Makhalanyane et al., 2013; McCarthy and Williams, 1992; Neilson et al., 2012). Consequently, *Streptomyces* has become a popular germplasm resource studied by experts and scholars worldwide. Between 2000 and 2021, a total of 129 new species of actinomycetes were discovered and documented across 35 desert environments globally. These *actinomycetes* have produced over 50 new compounds with potential applications in medicine, agriculture, and industry (Xie and Pathom-Aree, 2021). It has been found that *Streptomyces asenjonii* sp. nov. can produce new compounds Asenjonamides A-C (Abdelkader et al., 2018). This underscores the untapped potential of desert microorganisms and their secondary metabolites. As the predominant species of desert actinomycetes and a primary source of aminoglycosides, *Streptomyces* merits further exploration and utilization.

Natural aminoglycosides (AGs) are classified based on the primary sources of their strains. These include antibiotics derived from *Streptomyces* cultures, such as streptomycin and kanamycin, as well as those derived from Microsporum cultures, such as gentamicin and cissomycin (Becker and Cooper, 2013). Aminoglycosides offer significant advantages over other antibiotics; they are bactericidal rather than bacteriostatic for Gram-negative bacteria and can be effectively combined with other drugs, such as ampicillin, to enhance bacterial eradication. Furthermore, aminoglycosides are hypoallergenic and suitable for use in treating acute infections (Xie et al., 2011). Despite their critical role in clinical settings, the widespread use of these drugs has contributed to the emergence of drug resistance.

To discover new aminoglycosides, genome-guided metabolic potential mining serves as a crucial approach. For instance, crexazone 2 identified by genome mining of *Streptomyces* sp. CS057 (Prado-Alonso et al., 2024). Due to the highly polar and basic nature of aminoglycosides, these compounds lack chromophores and consequently exhibit no UV or fluorescence absorption, complicating their isolation and detection. To overcome these challenges, cation exchange resins are commonly employed for separation and purification. For example, Li et al. utilized a D4 cation exchange resin to isolate and purify kanamycin b, increasing its purity from 2.5 to 6% in the fermentation broth (Li et al., 2002). In terms of detecting kanamycin b, Li et al. applied a combination of one- and two-dimensional nuclear magnetic resonance (NMR) techniques to comparatively analyze the structural features of three aminoglycosides: etilmicin, 1,3-di-N-ethylgentamicin, and 1,6-di-N-ethylgentamicin, thereby establishing a method for the rapid and precise structural analysis of these antibiotics. The high sensitivity and selectivity of HPLC-MS/MS, along with the weakly basic characteristics of these compounds, contribute to a robust positive ion response during mass spectrometric analysis (Li et al., 2017; Yang et al., 2006). Additionally, an ultra-performance liquid chromatography-tandem mass spectrometry (UPLC-MS/MS) method has been developed for the simultaneous determination of nine aminoglycosides, including streptomycin, dihydrostreptomycin, neomycin, and gentamicin (Ma et al., 2020).

In this study, we conducted a comprehensive characterization and phylogenetic analysis on two novel strains, namely TRM 70351^T^ and TRM 70361^T^, which were isolated from poplar rhizomes collected in the Taklamakan Desert, Xinjiang, China. We further explored the metabolic potential of TRM 70351^T^ by detecting aminoglycoside signals using LC-MS, and then isolated and purified compounds daidzein and tridec-1-ene.

## Materials and methods

### Isolation and preserve

Soil samples were collected from poplar rhizomes in the Taklamakan Desert (37.813°N, 80.430°E), Xinjiang, China. The samples were then air-dried for 7 days. TRM 70351^T^ and TRM 70361^T^ were isolated on Gao’s agar containing sea salt (sea salt 33.3g, K_2_HPO_4_ 0.5g, MgSO_4_7H_2_O 0.5g, CaCO_3_ 1g, Agar 17g, H_2_O 1L of water Add a trace of salt 1ml before pouring the plate). The strains were preserved in tubes containing 20% glycerol or 20% milk, respectively.

### 16S *rRNA* gene phylogeny

The 16*S rRNA* gene was amplified from strains TRM 70351^T^ and TRM 70361^T^ using universal primers 27F (5′-AGAGTTTGATCCTGGCTC-3′) and 1492R (5′-CGGCTACCTTGTTACGACTT-3′) (Qingke Biotechnology, China) following the extraction of genomic DNA with a Bacterial DNA Isolation KitSequencing was performed by sanger sequencing using the 3730XL sequencing platform. The sequence was assembled using SeqMan software (available from DNA Star and accessible at https://www.dnastar.com/software/lasergene/seqman-ultra/). The complete 16S rRNA sequence was compared to the *16S rRNA* gene sequences of other species of *Streptomyces* in the EZbiocloud database, and the 16S rRNA sequence of the strain was stored in the GenBank/EMBL/DDBJ database (PQ758399, PQ758398). Phylogenetic trees were generated using three methodologies, namely neighbor-joining (NJ) (Saitou and Nei, 1987), maximum-likelihood (ML) (Felsenstein, 1981), and maximum-parsimony (MP) (Mount, 2008), which were facilitated by MEGA X software (Kumar et al., 2018). A multilocus sequence analysis (MLSA) tree was constructed by utilizing five housekeeping genes, namely *atpD*, *gyrB*, *recA*, *rpoB*, and *trpB*^1^ (Komaki, 2022). The genetic distance was conducted utilizing Kimura’s two-parameter model (Kimura, 1980). Bootstrap values were obtained in a test of tree topology stability with 1,000 replicates (Berner, 2009). Strain TRM 70351^T^ and TRM 70361^T^ genome was sequenced using the Illumina HiSeq 2000 platform (Hu et al., 2015). The genomic of the strains was assembled by using Abyss 2.0 (Jackman et al., 2017). The genome was carried out with the rapid annotations tool, facilitated by Prokka (Seemann, 2014). The digital DNA-DNA hybridization (dDDH) values were calculated using formula 2 on the GGDC website.^2^ The average nucleotide identity (ANI) was determined using OrthoANI, with default parameters (Yoon et al., 2017). Cross-checks such as ANIb, ANIm were also used in this study to better determine the new species status of the strains^3^ (Riesco and Trujillo, 2024). In this study, we evaluated the potential of TRM 70351^T^ to synthesize aminoglycosides using a comparative genomics approach to elucidate the biosynthetic potential of the strain (Blin et al., 2021).

### Phenotypic characterization

After 7 days of incubation in the International *Streptomyces* Program medium (Waksman, 1967), Gao’s medium (Kelly, 1964), PDA, NA, and Czapek-Dox Agar, the colony morphology of TRM 70351^T^ and TRM 70361^T^ was observed at 37°C. The morphological characteristics of the colonies were examined using optical microscopy (BX41, Olympus) and scanning electron microscopy (JSM-6610LV, JEOL) for 7 days. The colors of the colonies were established based on established color standards and nomenclature. The experiments using carbon sources were conducted according to the methodology outlined by Pridham and Gottlieb (1948) and Shirling and Gottlieb (1966). *Streptomyces* has a variety of physiological and biochemical characteristics, as previously describied (McCarthy and Williams, 1992; Pridham and Gottlieb, 1948). The growth capabilities of strains TRM 70351^T^ and TRM 70361^T^ were assessed across a temperature range of 10–55°C (specifically at 10, 12, 15, 20, 25, 28, 30, 37, 40, 45, 50, and 55°C) and a pH range of 4–12 (including pH values of 4, 5, 6, 7, 8, 9, 10, 11, and 12). Additionally, their tolerance to NaCl concentrations ranging from 0 to 10% (0, 1, 2, 3, 4, 5, 6, 7, 8, 9, and 10%, w/v) was evaluated using Gao’s broth as the basal medium. The production of peroxidase, urease, esterase, and catalase was tested utilizing the method described by Gerhardt et al. (1994). Determination of optimum conditions by observation and mycelial centrifugal weighing.

### Phenotypic and chemotaxonomic characterization

To study cell biomass, cells are grown in flasks with liquid Gause’s medium for 7 days at 37°C. Then, the cells are spun around at 12,000 rpm for 10 minutes (Centrifuge: cence-H1750R, Hunan) and then washed with distilled water twice. The Whole-cell sugars were analyzed using procedures developed by Kim (Freeze-dryer: BMC FD-1B-50+Beijing) (Kim and Goodfellow, 1999). Menaquinones were extracted from freeze-dried biomass and purified following the protocol established by Collins et al. (1977). Cellular fatty acid analyses were conducted in accordance with the protocol of the Sherlock Microbial ID System.^4^ Standard methods were used to determine the types of amino acids and total sugars in the cell wall hydrolysates (Li et al., 2019). Polar lipids were prepared according to Minnikin’s method, extracted (Spin Vapour Ankeyq N-1100), detected by two-dimensional thin-layer chromatography (Display laminate: TLC Silica gel 60), and analyzed with 10% molybdate ethanol (Zhang et al., 2018).

### TRM 70351 fermentation crude extract activity and HSQC-TOCSY detection

The uncontaminated seed liquid was inoculated into a pre-configured fermentation medium. One liter of each of the four mediums—namely millet medium, P4 medium, Streptomycin fermentation medium No. 1 (Han and Guirong, 2008) and Streptomycin fermentation medium No. 2 (Li et al., 2013) was selected, with an inoculum volume of 1%. This selection was made in conjunction with the literature on streptomycin fermentation and the growth state of the medium. The fermentation was conducted for 7 days at a temperature of 35^°^C. Another bottle of medium (200 mL) was used as a blank control. The samples were dried by freezing. After freezeing, the samples were soaked in 60% methanol, ultrasonicated, concentrated, and analyzed for activity using the Oxford cup method, while 5 mg of frozen samples were sent for HSQC-TOCSY to detect aminoglycosides (Bruker Ascend 500 MHz). Based on the activity and HSQC-TOCSY findings, Streptomycin fermentation medium No.1 of 70 L was selected for batch fermentation (fermenter:BIOTECH-100JS-7000, Shanghai) (Li et al., 2017).

### Isolation and identification of compounds

After fermentation, the crude product obtained after fermentation was redissolved in water and subsequently filtered through four layers of gauze. The resulting filtrate was adsorbent with D101 macroporous resin and D4 cation exchange resin. The D101 column was mixed with 80% methanol and the extract was concentrated in the methanol phase at low pressure. For the cation exchange resin column, elution was performed using a methanol:ammonia (3:1) elution and the extract was concentrated under reduced pressure in the alkaline aqueous. A total of 5 mg of the sample, along with standards of neomycin sulfate and streptomycin sulfate, were prepared by employing D_2_O as the solvent. The sample was diluted to a concentration of 5 μg/mL, and detection was performed using LC-MS/MS in an ESI scanning mode with positive ion detection (Ma et al., 2020; Yang et al., 2006).

In order to obtain additional compounds, the spreading agent was selected as dichloromethane:methanol, using the traditional separation method TLC. The spreading ratio for the spreading layer is determined by 1:1, 3:1, 5:1, 10:1, 20:1, 30:1, 40:1, and 50:1. Select sulfuric acid as the color development agent. The mass ratio of crude extracts to silica gel was 1:1, and according to the retention factor (Rf), dichloromethane:methanol 30:1 was selected as the eluent for the elution. A total of eight groups of samples were collected. The yield of compound 1 was obtained by concentrating sample 7. The concentrated samples 1–5 were utilized for subsequent elution, with a ratio of 5:1 between dichloromenthane and ethyl acetate. One group of 500 mL was selected, and the color developer was chosen to be sulfuric acid, Compound 2 was obtained by concentrating sample 4. The compounds were identified using mass spectrometry and nuclear magnetic resonance (NMR) techniques (Li et al., 2017). A comparative analysis was conducted to investigate the structures of the monomer compounds in conjunction with existing literature.

## Results

### Polyphasic classification and genetic characterization of strains TRM 70351^T^ and TRM 70361^T^

The *16S rRNA* gene sequence of strain TRM 70351^T^ showed that the strain was closely related to *Streptomyces chumphonensis* K1-2^T^ ( = JCM 18522^T^, 98.34%). Analysis of the phylogenetic tree and genome and *16S rRNA* gene sequences revealed that strain TRM 70351^T^ shared the same node with *S. chumphonensis* K1-2^T^ (Supplementary Figure S1). The *16S rRNA* gene sequence of strain TRM 70361^T^ showed a close relationship with *Streptomyces barkulensis* RC 1831^T^( = JCM 18754^T^, 98.15%) . The physiological tree of MLSA and genome and *16S rRNA* gene sequences analysis indicated that strain TRM 70361^T^ shared the same node with *S.carminius* TRM SA0054^T^. Both TRM 70351^T^ and TRM 70361^T^ are clustered with similar strains, suggesting that they are potential new species.

**FIGURE 1:**
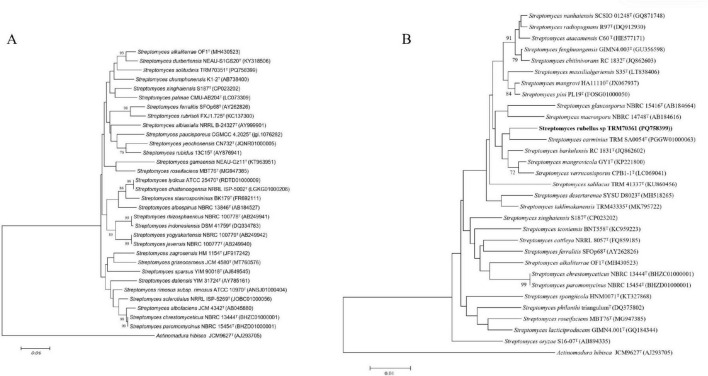
Neighbor-joining phylogenetic tree based on nearly complete *16S rRNA* gene sequences. **(A)** The relationships between strain TRM 70351. **(B)** TRM 70361, and the type strains of closely related *Streptomyces* species were analyzed. *Actinomadura hibisca* JCM 9627^T^ was used as the outgroup.

The analysis of the MLSA tree revealed that strain TRM 70351^T^ was situated in close proximity to *Streptomyces chumphonensis* K1-2^T^ (Figure 1). Based on the highest *16S rRNA* gene similarity, *16S rRNA* gene tree, MLSA results, and phylogenetic tree, the type strains of *S.chumphonensis* K1-2^T^ and *S.durbertensis* NEAU-S1GS20^T^ were selected for further comparisons with strain TRM 70351^T^. The results of the MLSA tree analysis indicated that strain TRM 70361^T^ was situated at the same node as *S. carminius* TRM SA0054^T^. Based on the highest *16S rRNA* gene similarity, *16S rRNA* gene tree, MLSA results, and phylogenetic tree, the type strains of *S.barkulensis* RC 1831^T^ and *S.carminius* TRM SA0054^T^ were chosen for further comparison with strain TRM 70361^T^ (Supplementary Figure S2).

All of the calculated ANI values and ANIb and ANIm were below the 95% threshold for determining distinct species (Supplementary Table S2). Moreover, all the estimated dDDH values were below the threshold for species differentiation (70%) (Chun et al., 2018),. The summary of these findings has been presented in Table 1. Based on the ANI and dDDH values, it appears that the strains TRM 70351^T^ and TRM 70361^T^ are novel *Streptomyces* species.

**TABLE 1:** Genomic homology analysis of strain TRM 70351 and strain TRM 70361 with similar strains.

Strain(genome accession number)	Size(bp)	DNA G+C content (mol%)	DNA-DNA relatedness(%)	Average nucleotide identify(%)
TRM 70351	5940221	73.69	–	–
*Streptomyces chumphonensis* K1-2 (GCA_014779715.1)	5823549	73.3	28.7	81.37
*Streptomyces alkaliterrae* OF1 (GCA_007097205.2)	6036367	72.0	21.7	78.94
*Streptomyces durbertensis* NEAU-S1GS20 (GCA_014156695.1)	5923645	72.4	21.6	79.29
*Streptomyces cattleya* NRRL 8057(GCA_000237305.1)	8092553	72.9	17.4	77.35
*Streptomyces xinghaiensis* S187 (GCA_000220705.2)	7137891	73.1	18.3	77.87
*Streptomyces rimosus subsp*. Rimosus ATCC 10970 (GCA_000331185.2)	9643891	72.0	17.2	77.73
*Streptomyces paromomycinus* NBRC 15454 (GCA_003865155.1)	9705398	72.0	17.0	77.80
*Streptomyces chrestomyceticus* NBRC 13444 (GCA_003865135.1)	9377418	72.0	17.1	78.00
*Streptomyces albospinus* NBRC 13846(GCA_014648695.1)	9206985	70.9	16.6	77.82
**Strain**	**Size(bp)**	**DNA G+C content(mol%)**	**DNA-DNA relatedness(%)**	**Average nucleotide identify (%)**
TRM 70361	6586496	73.42	–	–
*Streptomyces carminius* TRM SA0054 (GCA_002794255.1)	7200897	73.2	67.9	94.14
*Streptomyces barkulensis* RC 1831 (GCA_002843305.1)	6544873	72.9	33.1	84.19
*Streptomyces pini* PL19 (GCA_900114215.1)	6338888	73.0	33.5	84.57
*Streptomyces verrucosisporus* CPB1-1(GCA_017114865.1)	6063600	73.0	33.9	84.56
*Streptomyces radiopugnans* R97 (GCA_900110735.1)	6067124	73.4	35.7	84.75
*Streptomyces taklimakanensis* TRM 43335 (GCA_009709575.1)	6136714	72.8	33.0%	84.07

### Physiology, morphological characteristics

**FIGURE 2:**
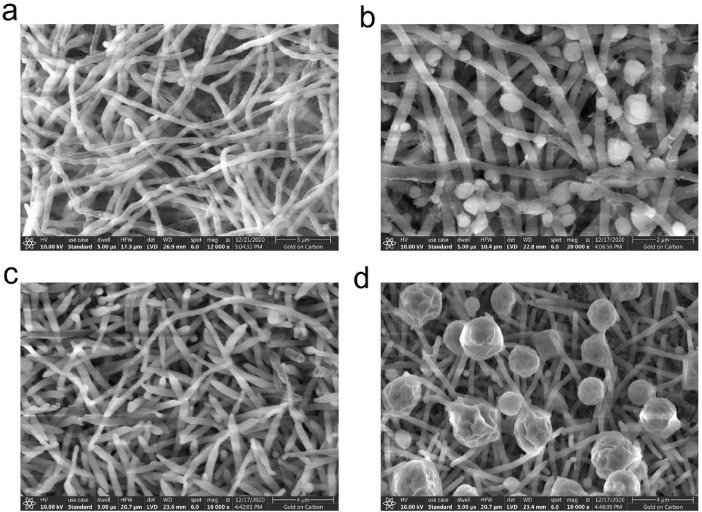
Scanning electron microscopy image of strain TRM 70351^T^ grown on Gao’s agar at 37°C for 7 days. **(A)** Mycelium of strain TRM 70351^T^. **(B)** Spores of strain TRM 70351^T^. **(C)** Mycelium of strain TRM 70361^T^. **(D)** Spores of strain TRM 70361^T^.

TRM 70351^T^ produced straight, lengthy chains of non-motile spores (Rectiflexibiles) with rough surfaces (Figures 2A,B), whereas TRM 70361^T^ has sporangia, each of which is rosebud-shaped, with hairs on the surface of the mycelium (Figures 2C,D). TRM 70351^T^ demonstrated excellent growth on ISP 1, 2, 4, Czapek’s and Gao’s medium (Table 2). The aerial mycelium and substrate mycelium of TRM 70351^T^ were developed without fragmentation, and the aerial mycelium exhibited a white to off-white appearance on all medium, while the substrate mycelium exhibited a white to yellow-white appearance. This strain has an optimal growth at pH 7–8. The optimal growth temperature ranged from 28to 40?. The growth occurred in the presence of 0–8% NaCl (w/v) as indicated in Table 2. TRM 70361^T^ demonstrated significant growth on both ISP 2, 4, and Gao’s medium. TRM 70361^T^ was developed without fragmentation of the aerial mycelium and substrate mycelium. The aerial mycelium appeared to be white to carmine on all media, and the substrate mycelium appeared to be white to yellow-white. The optimal pH ranges from 6 to 7. The optimal growth temperature was 30?. The growth was observed in the presence of 0-3% NaCl (w/v). The detailed physiological and biochemical properties are shown in Table 2 and in the species description.

**TABLE 2:** TRM 70351^T^,TRM 70361^T^ phenotypic characterization.

Characteristic	TRM 70351	*S.Chumphonens* K1-2	*S.durbertensis* NEAU-S1GS20	TRM 70361	*S.barkulensis* RC 1831	*S.carminius* TRM SA0054
Colony colour (Gauses medium)	White-yellow	White-yellow	White-yellow	carmine	off-white	carmine
Spore shape	Straight or flexuouschains	straight, long chains	Straight or fleuous chains	–	Straight or flexuous	–
Motility	–	–	–	–	–	–
Temperature range(?)	15-40	28-30	18-45	28-45	30-40	25-45
pH range	7-9	6-11	6-10	6-7	7 -10	5-10
NaCl (%, w/v)range	0-8	1-10	0-10	0-3	0-10	0-5
Gelatin liquefaction	+	+	+	+	+	–
Starch hydrolysis	+	+	+	+	+	+
Nitrate reduction	+	+	–	+	–	+
Urease production	–	–	+	–	–	–
Tween 20	+	+	+	+	+	+
Tween 40	+	+	+	+	+	+
Tween 60	+	+	+	+	+	+
Tween 80	+	+	+	+	+	+
**Utilization of:**						
D-Xylose	+	–	–	+	+	–
D-Glucose	+	+	+	–	+	+
D-Fructose	–	–	+	–	+	–
D-Mannose	+	–	+	–	+	+
L-Rhamnose	–	–	+	–	+	+
**Characteristic**	**TRM 70351**	***S.Chumphonens* K1-2**	***S.durbertensis* NEAU-S1GS20**	**TRM 70361**	***S.barkulensis* RC 1831**	***S.carminius* TRM SA0054**
**Chemical indicators:**						
cell-wall peptidoglycan.	LL-DAP	LL-DAP	–	LL-DAP	LL-DAP	LL-DAP
whole-cell sugars	Mannose, 2 unknown	glucose,ribose	glucose,ribose	glucose	glucose,ribose	Glucose
polar lipids	DPG,PE,PI,PIM	PE,DPG,PG,PI,PIM	DPG,PE,PI,NPG(two unidentified phospholipids)	PIM,PI,PE	DPG,PE,PI,PIM(one unidentified phospholipids)	DPG,PME,PE,PC, PI,PIM(one unidentified phospholipids)
menaquinones	MK6,MK7,MK-10(H2),MK-10(H4)	MK9-(H6),MK9-(H8)	MK-9(H4),MK-9(H6)	MK-6MK-6(H2),MK-8MK-9(H8),MK-10(H6)	MK-9(H4),MK-9(H6),MK-9(H8),MK-9(H2),MK-9,MK-8,MK-10,MK-10(H6),MK-10(H8)	MK-8(H4),MK-9(H6),MK-9(H8)
fatty acids	C_8:03OH_,anteiso-C_15:0_,iso-C_16:0_,C_16:0_,anteiso-C_17:0_,Sum In Feature 6,Summed Feature 6	Anteiso-C_15:0_,iso-C_16:0_,iso-C1_5:0_	iso-C_16:0_,iso-C_17:0_,Anteiso-C1_7:0_,C_16:0_	C_8:03OH_,anteiso-C_15:0_,16:1 iso H,iso-C_16:0_,anteiso-C_17:0_,Sum In Feature 6,Summed Feature 6	iso-C_14:0_, C_14:0_,iso-C_15 : 0_, antiso-C1_5:0_,C_15:1_ B,C_15:0_,iso-C1_6:1_,Hiso-C_16:0_, C_16:1_ω9c,C_16:0_,methyl C_16:0_, antiso-C_17 :1_,iso-C_17:0_,C17 : 1ω9 cantiso-C_17:0_,iso-C18:1H, iso-C1_8 : 0_,C_18:1_ω9c, C_18:0_,C1_4_ : 1ω9t/9c,C_18:1_ω11c/9t/6t	isoC_16:0_,iso-C_16:1_,Ganteiso-C_17:0_,anteiso-C_15:0_, C_16:0_,anteiso-C_17:1_ω9c

+, Positive; -, Negative.

### Chemical composition characteristics

TRM 70351^T^ contained LL-diaminopimelic acid in its cell-wall peptidoglycan and mainly contained mannitol as whole-cell hydrolysates, along with two unidentified hydrolysates. As illustrated in Supplementary Figure S3, the major polar lipids of TRM 70351^T^ included phosphatidylglycerol, phosphatidylethanolamine, phosphatidylinositol, and phosphatidylinositol mannosides. The dominant menaquinones in TRM 70351^T^ were MK6, MK7, MK-10(H2), and MK-10(H4). The principal cellular fatty acids were identified as anteiso-C_15:0_, iso-C_16:0_, C_16:0_, and anteiso-C_17:0_, along with summed feature 6 (Table 2; Supplementary Table S3). TRM 70361^T^ contained LL-diaminopimelic acid in the cell-wall peptidoglycan and primarily contained glucose as whole-cell hydrolysates. The major polar lipids of TRM 70361^T^ comprised phosphatidylethanolamine, phosphatidylinositol, and phosphatidylinositol mannosides, as depicted in Supplementary Figure S3. The predominant menaquinones present in TRM 70361^T^ were identified as MK6, MK6(H2), MK-8, MK-9(H8), and MK-10(H6). The major cellular fatty acids identified in Table 2. It is C8:0 3OH, anteiso-C_15:0_, 16:1 iso H, iso-_*C16:0*_, and anteiso-C_17:0_ (Table 2; Supplementary Table S3).

### Analysis of the genomic metabolic capacity and biosynthetic gene cluster of TRM 70351^T^ is conducted

TRM 70351^T^ has a genome size of 5,940,221 bp, a coding sequence count of 5,223, and a G+C content of 73.69 mol%. A total of 36 biosynthetic gene clusters were predicted within the TRM 70351^T^ genome (Table 3). These include one amglyccycl, betalactone, hglE-KS, lanthipeptide-class-iii, lassopeptide, NRPS-independent-siderophore, and T2PKS. Additionally, there are two NRPS biosynthetic gene cluster, two lanthipeptide-class-i biosynthetic gene cluster, three NRPS-like biosynthetic gene cluster, three terpene types, five T1PKS types, and thirteen other heterozygous types in total. Among these, gene clusters 6.1 and 8.2 show 100% similarity to known biosynthetic gene clusters, indicating that the strain is capable of producing the corresponding secondary metabolites. However, antiSMASH did not predict gene clusters 3.3 and 4.1 because they were not similar to known biosynthetic gene clusters groups. It is proposed that these gene clusters may indeed produce natural products that differ in both structure and synthesis mechanisms from those of known secondary metabolites. Clusters 1.4, 1.7, 2.3, 3.1, 3.2, 5.1, 5.3, 12.1, and 13.1 exhibit a high degree of similarity (>50%) to known gene clusters, indicating a greater likelihood of producing corresponding compounds. It is notable that gene cluster 1.4 is predicted to produce ribosome-synthesized and post-translationally modified peptides (RiPPs), such as streptamidine, with a similarity of 75%. Streptamidine is recognized as a structurally diverse natural product with a wide range of biological activities, and is recognized as a structurally diverse natural product. Gene cluster 3.1 corresponds to antimycins (93%) belonging to the NRPS class, which possess antifungal, insecticidal, and antibiotic properties. Gene cluster 5.3 is predicted to correspond to a type 1 polyketide synthase compound, camporidine, which exhibits 60% similarity. Camporidine is a polyketide alkaloid with anti-inflammatory properties. Gene cluster 13.1 is predicted to yield a non-ribosomal peptide synthase (NRPS) compound, bohemamine, with a 50% similarity to NRPS. Bohemamine is classified within the alkaloid group and exhibits a diverse- range of biological activities, including antibacterial properties against gram-negative bacteria. Furthermore, there are a substantial number of antibiotic-like gene clusters with less than 50% similarity, including kinamycin, azicemicin B, and formicamycins A-M, among various others. Additionally, TRM 70351^T^ holds significant potential for the discovery of secondary metabolites.

**TABLE 3:** Prediction of secondary metabolite biosynthesis gene cluster of TRM 70351^T^.

Region	Type	From	To	Most similar known cluster	Similarity
1.1	T1PKS,NRPS-independent- siderophore	33,127	79,260	kinamycin	13%
1.2	LAP,thiopeptide	171,277	206,384	ulleungmycin	5%
1.3	lanthipeptide-class-i,NRPS, NRPS-like,T1PKS	234,387	303,249	enduracidin	10%
1.4	RiPP-like	364,879	376,267	streptamidine	75%
1.5	NRPS-like	377,145	420,252	paulomycin	9%
1.6	terpene	535,952	557,344	hopene	30%
1.7	terpene,crocagin	675,154	717,581	isorenieratene	87%
2.1	betalactone	22,854	46,984	aurantimycin A	5%
2.2	lanthipeptide-class-i	86,374	110,191	BD-12	14%
2.3	NRP-metallophore,NRPS	285,207	350,253	mirubactin	78%
2.4	thioamide-NRP,ectoine, T3PKS	618,922	683,299	azicemicin B	13%
3.1	NRPS,T1PKS	300,121	352,721	antimycin	93%
3.2	lanthipeptide-class-iii	590822	613,410	SapB	75%
3.3	terpene	689,050	710,672	–	–
4.1	NRPS-like,betalactone	35,808	86,488	–	–
4.2	NRPS-like	109,523	151,301	marineosin A/marineosin B	31%
4.3	NRPS-like,butyrolactone	157,152	199,110	diastaphenazine/ izumiphenazine C	35%
4.4	NRPS	268,148	333,218	omnipeptin	11%
5.1	T2PKS	80,651	153,193	griseusin	53%
5.2	T1PKS	386,410	432,130	colibrimycin	3%
5.3	T1PKS	517,686	549,744	camporidine A/camporidine B	60%
6.1	terpene	338,010	360,262	geosmin	100%
6.2	NRPS-like,betalactone	391,448	433,406	longicatenamide B/longicatenamide C/longicatenamide A/longicatenamide D	15%
7.1	butyrolactone,T1PKS	42,071	91,641	chlorothricin/deschlorothricin	4%
7.2	T2PKS,butyrolactone	252,599	291,677	lactonamycin	10%
8.1	NRPS-independent- siderophore	85,255	101,842	nonactin/monactin/dinactin/trinactin/tetranactin	33%
8.2	T3PKS,ectoine	147,419	194,536	ectoine	100%
9.1	lassopeptide	19,875	42,468	mycotrienin I	7%
9.2	T1PKS	167,413	215,023	formicamycins A-M	4%
10.1	lanthipeptide-class-i	156,703	180,865	colibrimycin	5%
11.1	T1PKS	24,239	77,557	pyrrolomycin A/pyrrolomycin B/ pyrrolomycin C/pyrrolomycin D	40%
12.1	amglyccycl	71,861	95,511	streptomycin	55%
13.1	NRPS	18,720	65,952	bohemamine A/bohemamine B/bohemamine C	50%
14.1	hglE-KS	8,783	62,811	A33853	17%
16.1	NRPS-like	3,773	39,108	indigoidine	27%
18.1	T1PKS	1	11,057	argimycin PI/argimycin PII/nigrifactin/argimycin PIV/argimycin PV/argimycin PVI/argimycin PIX	10%

**FIGURE 3:**
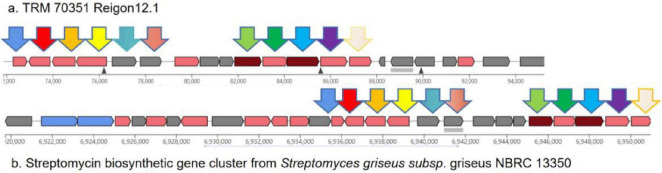
Comparison of gene cluster 12.1 predicted genes for TRM 70351^T^ strain. **(A)** TRM 70351 Reigon12.1. **(B)** Streptomycin biosynthetic gene cluster from *Streptomyces griseus* subsp. Griseus NBRC 13350.

The gene cluster 12.1 is predicted to be an aminoglycoside biosynthetic gene cluster for streptomycin, exhibiting 55% similarity (see Figure 3). Aminoglycosides have a broad range of antimicrobial and bacteriostatic properties. Upon further examination of this gene cluster, two core genes, nine supplementary genes, and various other types of coding genes were identified within the biosynthetic gene cluster. Using BLAST to compare the gene functions of core genes and additional genes (Figure 3), it was found that the core genes have the same function as the genes in the streptomycin biosynthetic gene cluster of *Streptomyces griseus*, and they are both responsible for encoding dTDP-dihydrostreptose-streptidine-6-phosphate and alkaline phosphatase, the similarities are 98.18 and 97.84%. In addition, there are 9 additional genes, among which inosamine-phosphate amidinotransferase 1 and beta-ketoacyl-ACP reductase are unique to strain TRM 70351^T^, with similarities of 100 and 96.15%. The remaining genes have the same functions as those in the streptomycin biosynthetic gene cluster of *Streptomyces griseus*, including dTDP-4-dehydrorhamnose 3,5-epimerase, dTDP-4-dehydrorhamnose reductase, glucose-1-phosphate thymidylyltransferase, etc., with a similarity of 98.50, 97.04, and 98.59%. TRM 70351^T^ were transporter-related genes: ABC transporter ATP-binding protein and ABC transporter ATP-binding protein/permease. Given that TRM 70351^T^ has the core genes for streptomycin synthesis, as well as the synthesis genes for related aminotransferases and other key enzymes, it is speculated that this strain is extremely Streptomycin or structurally similar compounds may be synthesized.

### Selection of aminoglycoside fermentation media based on activity and spectral signals

Based on the antiSMASH prediction indicating the presence of the streptomycin biosynthetic gene cluster in the strain, four fermentation media were selected for HSQC-TOCSY analysis. Additional media were chosen based on activity screening results. The streptomycin fermentation samples showed significant activity against *Staphylococcus aureus*, *Klebsiella pneumoniae*, *Salmonella*, *Acinetobacter baumannii*, *Pseudomonas amylovora*, and *Pseudomonas aeruginosa*. In contrast, millet fermentation samples exhibited activity against *Enterococcus faecalis*, *Escherichia coli*, and *Klebsiella pneumoniae*. The ISP4 fermentation medium showed efficacy against Erwinia amylovora, while streptomycin fermentation medium No.2 (Li et al., 2013) demonstrated activity against *Staphylococcus aureus*, as illustrated in Supplementary Table S1 and Supplementary Figure S4. ISP4, known for its low-nutrient composition, did not exhibit aminoglycoside region signals post-fermentation, unlike the other three fermentation media samples. Considering both effectiveness and spectral signals, streptomycin fermentation medium No.1 (Han and Guirong, 2008) was chosen for the batch fermentation of TRM 70351^T^.

### Detection and characterization of metabolites

**FIGURE 4:**
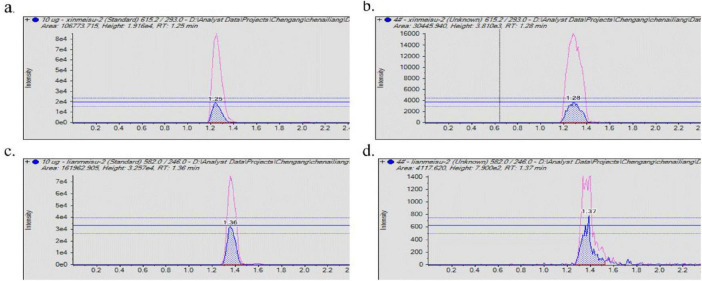
LC-MS/MS of alkaline aqueous extracts and standards. **(A)** Neomycin sulfate LC-MS/MS results. **(B)** TRM 70351 LC-MS/MS results. **(C)** Streptomycin sulfate LC-MS/MS results. **(D)** TRM 70351 LC-MS/MS results. The red peaks in the graph represent the response of quantitative ions and the blue peaks represent the response of qualitative ions.

The analysis of alkaline aqueous-phase extracts from the fermentation medium of Streptomycin No. 1 using the HSQC-TOCSY assay identified aminosugar signals in the region of δH 3.0-4.0 ppm andδC 45.0-55.0 ppm, as depicted in Figure 4. The HSQC-TOCSY region summary plots for the reference compounds, streptomycin sulfate and neomycin sulfate, are detailed in Figure 4. LC-MS/MS analysis was performed on standard and TRM 70351^T^ samples to determine the molecular weights of meomycin (615.0) and streptomycin (582.0) (see Figure 5). The targeted detection of TRM 70351^T^ revealed signals for neomycin (615.0, 160.9, and 293.0 m/z) and streptomycin (582.0, 263.0, and 246.0 m/z), confirming the presence of these aminoglycosides. In conclusion, it was estabilished that strain TRM 70351^T^ harbors neomycin and streptomycin.

Compounds 1 and 2 were isolated through conventional silica gel columns chromatography (Supplementary Figure S5), In this study, we mainly explored the large polar substances, so we chose methanol for the pre-extraction. Their structures were elucidated by NMR analysis and literature comparison. The pertinent data are presented belows. Compound 1, depicted by a hydrocarbon spectrum in Supplementary Figure S4, consists of light yellow crystals, molecular formula: C_15_H_10_O_4_. soluble in DMSO.^1^H NMR (500 MHz, DMSO-d6) δ 10.77 (s, ^1^H), 9.55 (s, ^1^H), 8.28 (s, ^1^H), 7.97 (d, *J* = 8.74 Hz, ^1^H), 7.39 (d, *J* = 8.43 Hz, ^2^H), 6.94 (dd, *J* = 8.76, 2.23 Hz, ^1^H), 6.86 (d, *J* = 2.23 Hz, ^1^H). = 8.43 Hz, 2H), 6.94 (dd, *J* = 8.76, 2.23 Hz, 1H), 6.86 (d, *J* = 2.23 Hz, 1H), 6.82 (d, *J* = 8.47 Hz, 2H). After conducting a literature review, the spectral data of this compound aligned perfectly with it, leading to the identification of compound as daidzein (Soidinsalo and Wähälä, 2004) (a soybean glycoside) with the structural formula depicted in Supplementary Figure S6.

Compound 2 is a colorless oil that exhibits solubility in petroleum ether, chloroform, and other similar solvents. Molecular formula C_13_H_26_. ^1^H-NMR (500 MHz, Chloroform-d)δ5.03-4.90 (m, 1H), 2.04 (q, *J* = 7.31, 7.17, 7.17 Hz, 1H), 1.37 (t, *J* = 7.32, 7.32 Hz, 1H), 1.30 (t, *J* = 12.28, 12.28 Hz, 1H), 1.28 (s, 4H), 1.26 (s, 9H), 1.21 (s, 1H), 1.18 (s, 3H), 0.91-0.78 (m, 4H). After literature review, the spectral data of the compound was in complete agreement with it, so the compound was identified as tridec-1-ene (Neipihoi et al., 2021) with the structural formula shown in Supplementary Figure S7.

### Description of *Streptomyces solitudinis* sp. nov

*Streptomyces solitudinis* (solitudin’is N.L. masc. adj, the strain is a new species from the *Taklamakan Desert* in China, where desert means solitude).

*Streptomyces solitudinis*, a Gram-positive *actinomycete*, is an aerobic, mesophilic microorganism that forms straight or flexuous chains of spores with rough surfaces and lacks motility. It displays vigorous growth on ISP media 1, 2, 4, as well as Czapek’s and Gao’s medium. The species thrives optimally at temperatures ranging from 15 to 40^°^C and pH levels between 7 and 9. Growth is supported in the presence of 0 to 8% NaCl (w/v). It metabolizes D-Xylose, D-Glucose, and D-Mannose, while showing no utilization of D-Fructose or L-Rhamnose. Nitrate undergoes conversion to nitrite with gelatin liquefaction showing variability but stability starch hydrolysis demonstrating positivity, and urease production exhibiting negativity. The cell wall’s peptidoglycancomprises LL-diaminopimelic acid, while mannitol and two unidentified hydrolysates constitute the whole-cell sugars. The primary phospholipids consist of diphosphatidylglycerol, phosphatidylethanolamine, phosphatidylinositol, and phosphatidylinositol mannosides. The prevalent menaquinones include MK6, MK7, MK-10(H2), and MK-10(H4). Major fatty acids detected are C_8:0 3OH_, anteiso-C_15:0_, iso-C_16:0_, C_16:0_, and anteiso-C_17:0_.

The type strain for *Streptomyces solitudinis* sp. nov. is TRM 70351^T^ ( = CCTCC AA 2020049^T^ = LMG 32404^T^), was recovered from China.

### Description of *Streptomyces rubellus* sp. nov

*Streptomyces rubellus* (*rubellus* N.L. masc. adj, the strain was red in color and resembled rubella in the electron micrographs, and was named *Streptomyces rubellus* based on the color morphology).

*Streptomyces rubellus*, a Gram-stain-positive *actinomycete* is an aerobic and mesophilic organism that is non-motile and lacks spores. It thrives on various media including ISP1, ISP2, ISP4, NA, and Gao’s medium. The species metabolizes D-Xylose exclusively, while showing no utilization D-Glucose, D-Mannose, D-Fructose, or L-Rhamnose. Nitrate is enzymatically reduced to nitrite. Optimal growth transpires within a temperature ranges of 28 to 45^°^C, and within a pH range of 6–7. Growth is sustained in the presence of NaCl concentrations ranging from 0 to 3% (w/v). The liquefaction of gelatin exhibits variablility but remains stable, whereas starch hydrolysis shows a positive response, however, urease production is negative. LL-diaminopimelic acid is detected in the cell wall, with glucose identified as the predominant whole-cell sugar. The primary phospholipids include phosphatidyl ethanolamine, phosphatidylinosito, and phosphotidylinositol mannosides. The main menaquinones present are MK6, MK6(H_2_), MK-8, MK-9(H_8_) and MK-10(H_6_). Predominant fatty acids consist of C_8:0 3OH_, anteiso-C_15:0_, 16:1 iso H, iso-C_16:0_ and anteiso-C_17:0_.

The type strain for *Streptomyces rubellus* sp. nov. is TRM 70361^T^( = CCTCC AA 2020043^T^ = JCM 35793^T^), was recovered from China.

## Discussion

In this investigation, two novel strains of *Streptomyces* species, namely TRM 70351^T^ and TRM 70361^T^, were discovered in rhizosphere soil samples collected from *Populus euphratic*a in the Taklimakan Desert, Xinjiang, China. Through contemporary taxonomic techniques, their genotypic, phenotypic, and chemical profiles were delineated, leading to their designation as *Streptomyces solitudinis* and *Streptomyces rubella*, correspondingly. Notably, the strain TRM 70351^T^ exhibited the production of aminoglycoside secondary metabolites during fermentation.

The DNA G+C content of TRM 70351 is 73.69 mol%, whereas TRM 70361^T^ exhibits a G+C content of 73.42%. Strains closely related to TRM 70351 include *Streptomyces chumphonensis* K1-2^T^ (Yu et al., 2018), *Streptomyces alkaliterrae* OF1^T^ (Świecimska et al., 2020), and *Streptomyces durbertensis* NEAU- S1GS20^T^ (Yu et al., 2018). There strains were studied: K1-2^T^, collected from marine environments in Thu Phuong Province, Thailand; OF1^T^, isolated from alkaline soil near a meteoric alkaline soda lake in India; and NEAU- S1GS20^T^, obtained from saline soil in Heilongjiang Province, northeastern China. Related strains included *Streptomyces barkulensis* RC 1831^T^ (Ray et al., 2014) and *Streptomyces carminius* TRM SA0054^T^ (Wang et al., 2018). RC 1831^T^ was isolated from sediments at a fish dumpsite near the brackish Chilika Lake in Balkur village, Kulda district, Orissa, India; while TRM SA0054^T^ was sourced from various locations, all of which were bitter ginseng roots in Alar, Xinjiang. Despite their disparate origins, both strains were sourced from alkaline environments, indicating shared survival conditions that underpin their similarities (Smithburn, 1936).

Preliminary statistics indicate the discovery of 155 new natural products from *streptomycetes* isolated in extreme environments like the deep sea, deserts, volcanoes, and polar regions during the period from 2009 to 2020 (Duan et al., 2022). *Streptomycetes* are also known as the primary sources of aminoglycosides, exemplified by streptomycin from *S. griseus* (Rao, 1948) and chloramphenicol from *S. hygroscopicus* (Tabakov et al., 1994). The identification of new *streptomycetes* from desert environments holds significant research value. This study utilized Mass spectrometry to analyze strain TRM 70351^T^, confirming its production of aminoglycosides such as neomycin and streptomycin. Furthermore, besides the isolation tridec-1-ene and daidzein, both known for their beneficial effects in related research, the study also explored the mining of other compounds. Tridec-1-ene shows promise as a chemopreventive agent by potentially activating Nrf2/ARE-mediated phase II enzymes (Bhat et al., 2022). Additionally, daidzein demonstrates notable antimicrobial properties (Saeed Kotb et al., 2024).

## Conclusion

In this investigation, two novel species of *Streptomyces*, namely strains TRM 70351^T^ and TRM 70361^T^ originating from the Taklamakan Desert, were characterized. The metabolic capacity for aminoglycoside compounds in strain TRM 70351 was explored, leading to the isolation of the isolation of the bioactive compounds tridec-1-ene and Daidzein using contemporary extraction methods. This discovery has significantly augmented the repository of strains for aminoglycoside exploration.

## Data Availability

The original contributions presented in the study are publicly available. This data can be found at: https://www.ncbi.nlm.nih.gov/, accession numbers PQ758399 and PQ758398; genome accession numbers PRJNA1068773 and PRJNA1068755.
