# Supplementary material for: Isolated from Populus euphratica rhizosphere soil, and mining their metabolites
Source: Front Microbiol. 2025 Feb 19;16:1530786. doi: 10.3389/fmicb.2025.1530786 (PMC11881777; doi:10.3389/fmicb.2025.1530786)
Supplement: Supplementary file 1 [file Supplementary_file_1.docx]

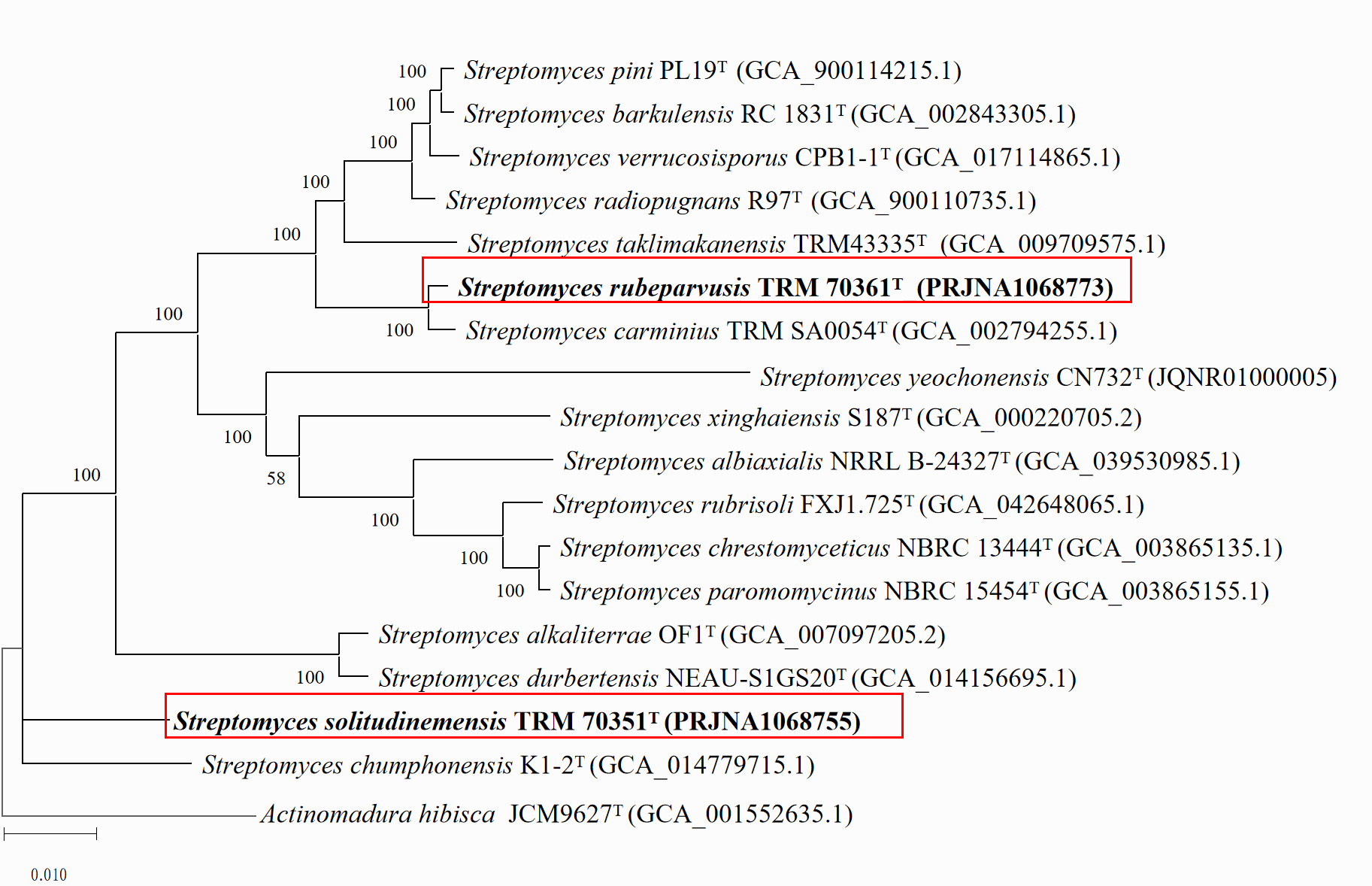


Support Fig 1 Construction of phylogenetic evolution tree based on core genome


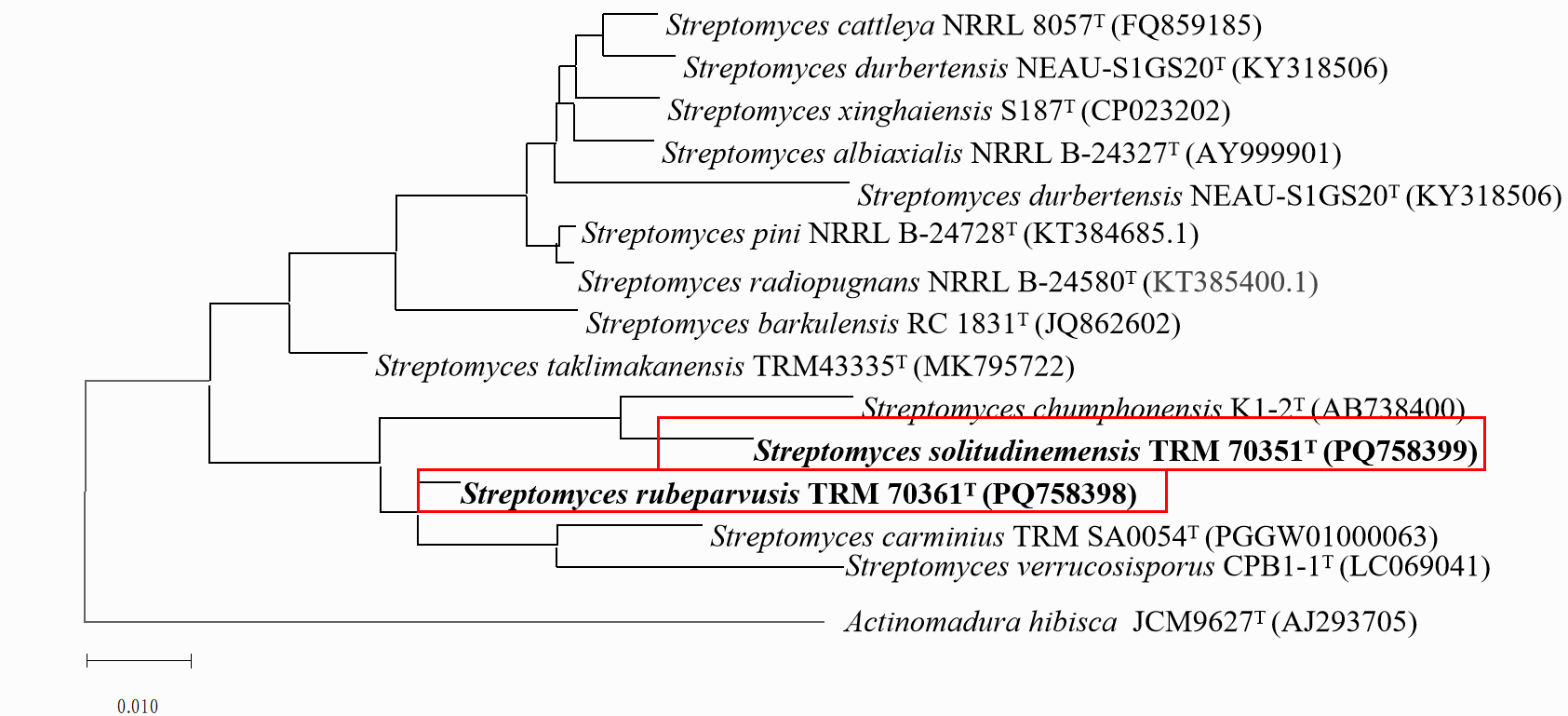


Support Fig 2 NJ tree based on the concatenated *atpD-gyrB-recA-rpoB-trpB* gene sequences showing the phylogenetic relationships the phylogenetic position of strain TRM 70351,TRM 70361 among related taxa.


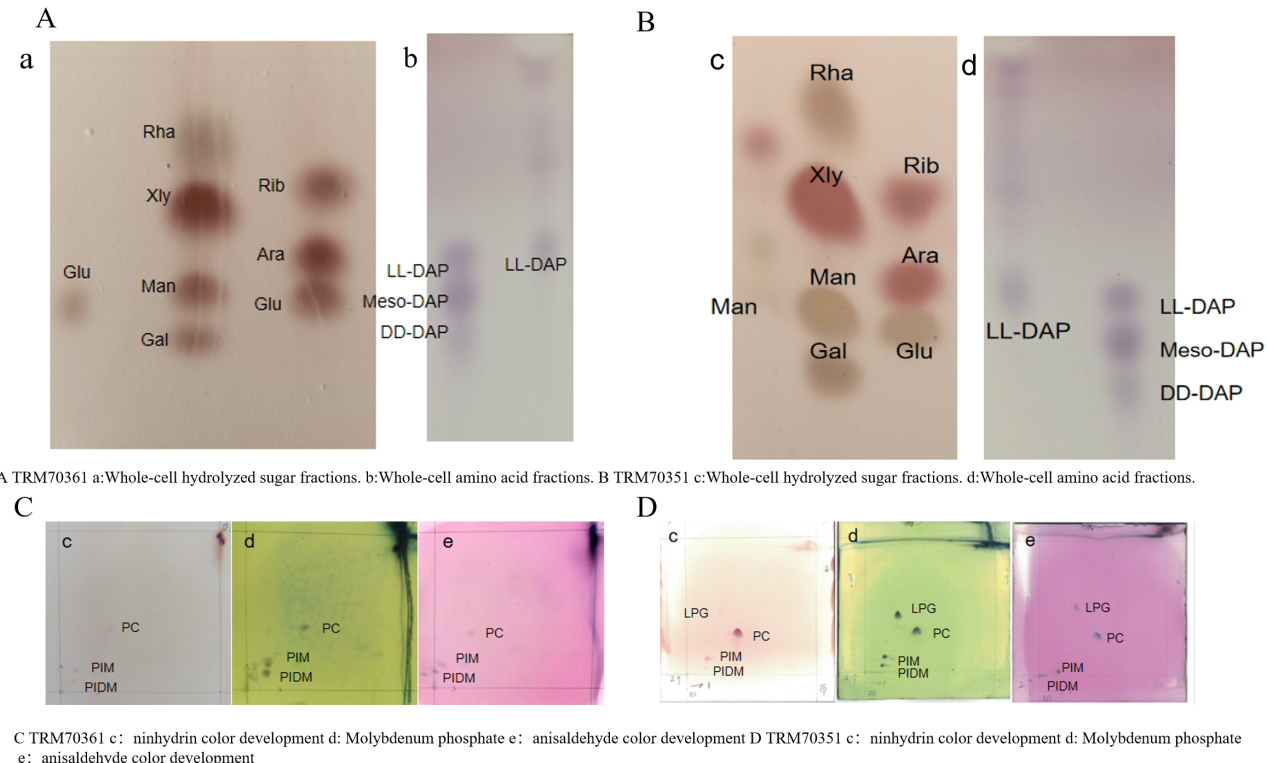
Support Fig3 Chemical specifications for TRM 70351 and TRM 70361.


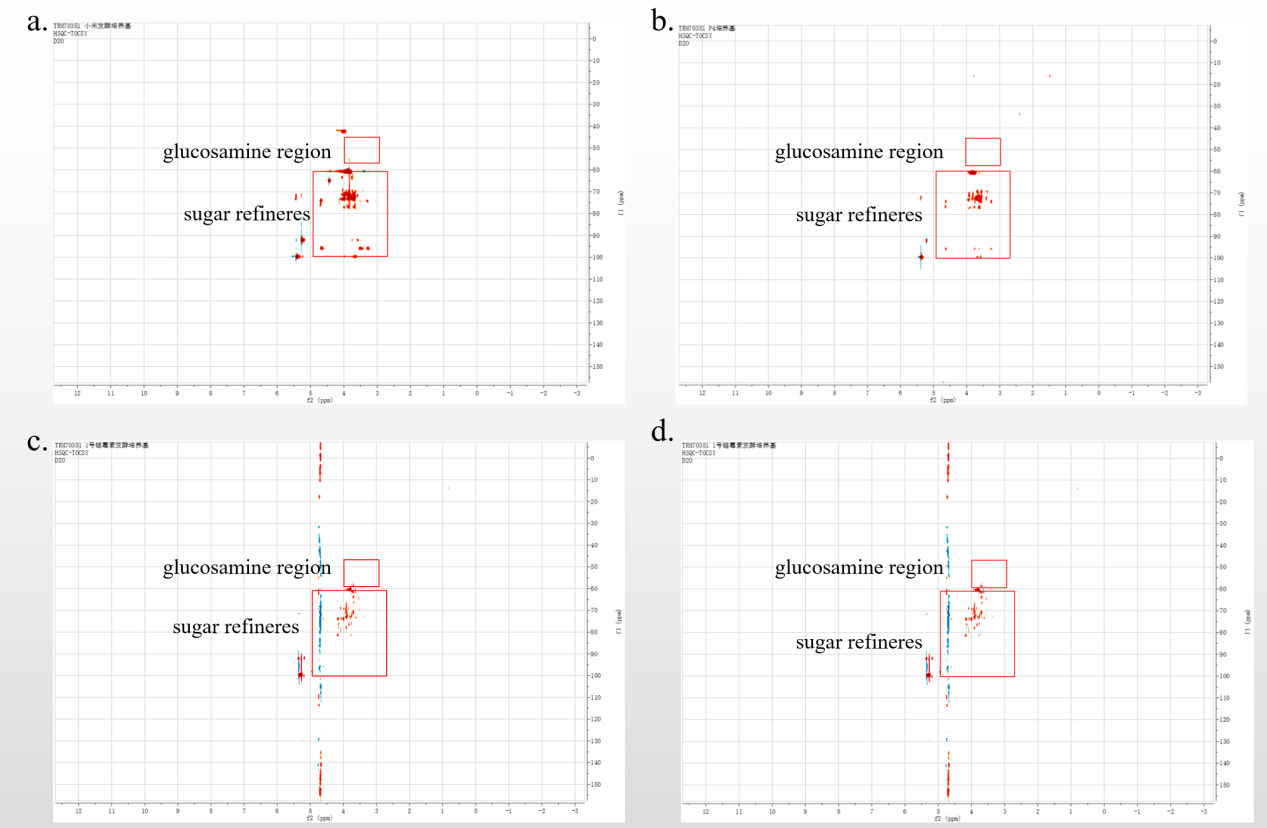


Support Fig4 HSQC-TOCSY was performed on crude samples of TRM 70351 in different medium


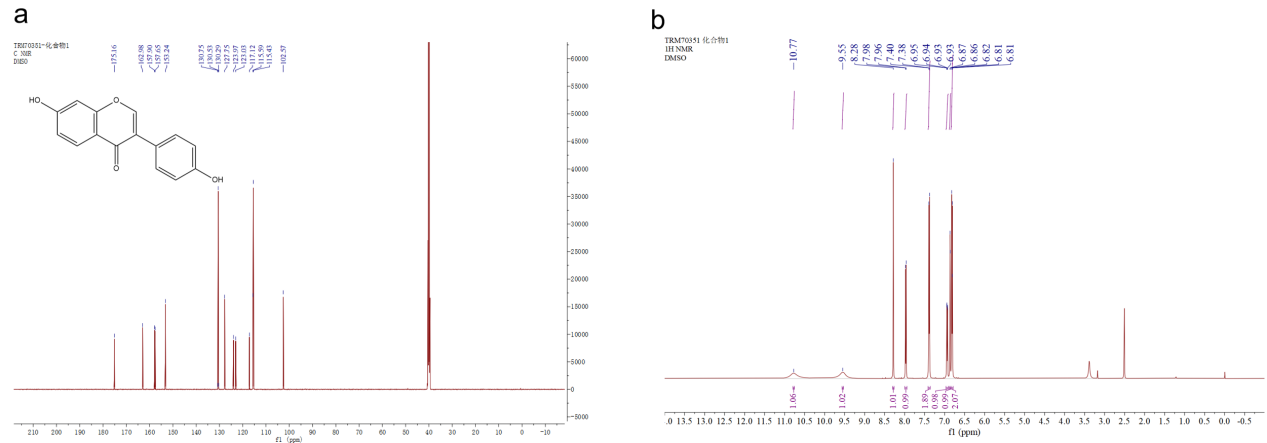

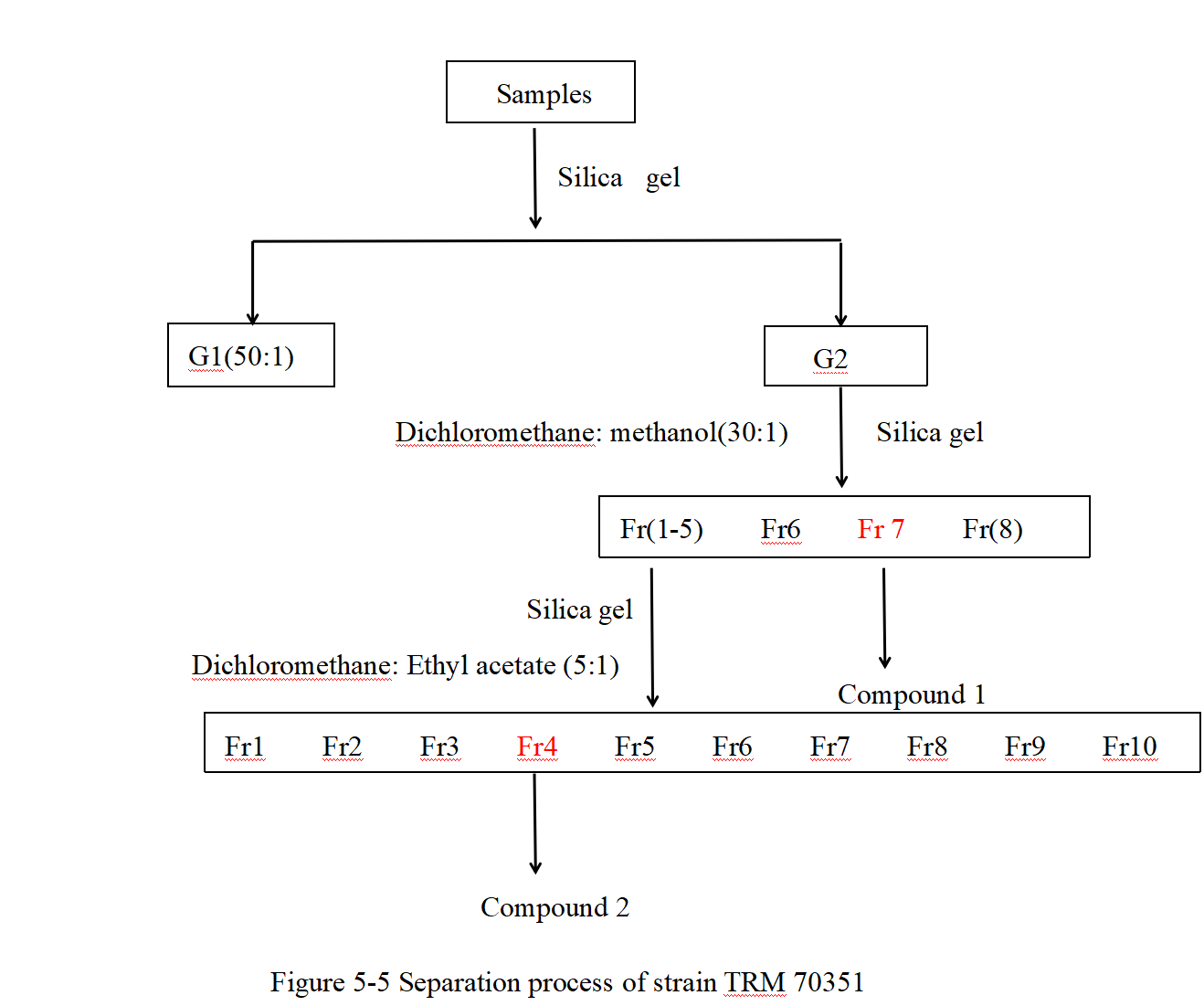
Note:a millet medium;b P4 medium;c Streptomycin fermentation medium No.1;d Streptomycin fermentation medium No.2

Support Figure 5 separation process of strain TRM 70351

Support Fig 6 NMR spectra of the compound daidzein

Note:NMR carbon spectrum of daidzein b Nuclear magnetic hydrogen spectrum of daidzein


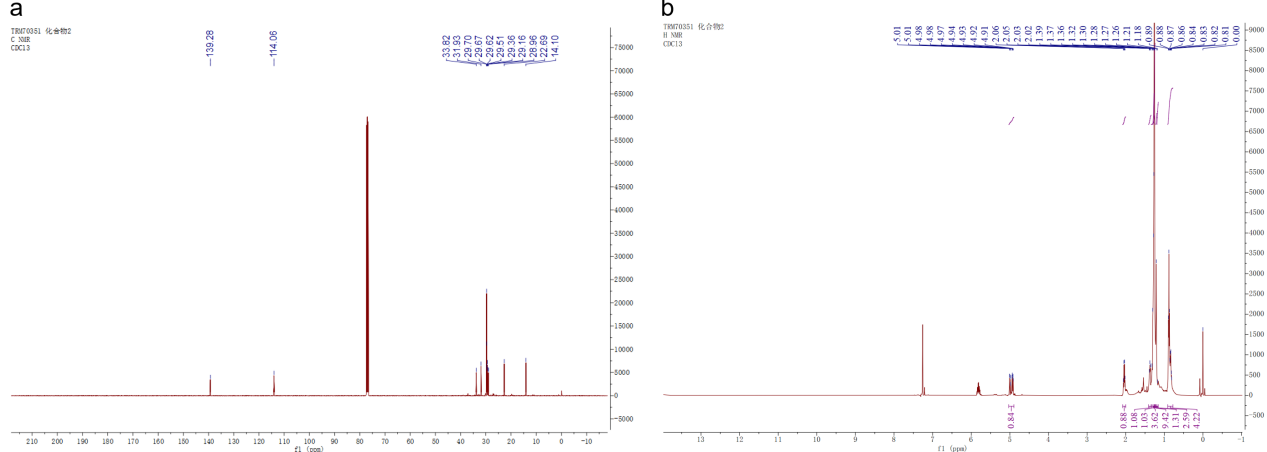


Support Fig 7 NMR spectra of the compound tridec-1-ene

Note:NMR carbon spectrum of tridec-1-ene b Nuclear magnetic hydrogen spectrum of tridec-1-ene
