# Supplementary material for: Isolated from Populus euphratica rhizosphere soil, and mining their metabolites
Source: Front Microbiol. 2025 Feb 19;16:1530786. doi: 10.3389/fmicb.2025.1530786 (PMC11881777; doi:10.3389/fmicb.2025.1530786)
Supplement: Supplementary file 2 [file Supplementary_file_2.docx]

| Support table 1 detection of bacteriostatic activity of four fermentation media TRM 70351 | | | | |
| --- | --- | --- | --- | --- |
| target bacterium | Millet fermentation medium | P4 fermentation medium | Streptomycin fermentation medium No.1 | Streptomycin fermentation medium No.2 |
| *S. aureus* | 0 | 0 | 12.21 mm | 19.51 mm |
| *E. faecium* | 12.85 mm | 0 | 0 | 0 |
| *E. coli* | 12.71 mm | 0 | 0 | 0 |
| *S. castellani* | 0 | 0 | 0 | 0 |
| *K.pnenmoniae* | 11.14 mm | 0 | 14.54 mm | 0 |
| *Salmonella* | 0 | 0 | 11.67 mm | 0 |
| *A.baumannii* | 0 | 0 | 15.05 mm | 0 |
| *E.amylovora* | 0 | 16.87 mm | 9.56 mm | 0 |
| *P.aeruginosa* | 0 | 0 | 15.26 mm | 0 |
| Note: oxford cup diameter 7 mm | | | | |

Support table 2 ANIb, ANIm values of strain TRM 70351 and strain TRM 70361 with their similar strains

| ANIm | Strains | *Streptomyces chumphonensis* K1-2 | TRM 70351 | *Streptomyces alkaliterrae* OF1 |
| --- | --- | --- | --- | --- |
|  | *Streptomyces chumphonensis* K1-2 | - | 86.06 | 85.64 |
|  | TRM 70351 | 86.06 | - | 85.71 |
|  | *Streptomyces alkaliterrae* OF1 | 85.71 | 85.71 | - |
| ANIb | *Streptomyces chumphonensis* K1-2 |  | 80.65 | 77.74 |
|  | TRM 70351 | 81.36 |  | 78.59 |
|  | *Streptomyces alkaliterrae* OF1 | 77.89 | 78.59 |  |
| ANIm | Strains | *Streptomyces carminius* TRM SA0054 | *Streptomyces barkulensis* RC 1831 | TRM 70361 |
|  | *Streptomyces carminius* TRM SA0054 | * | 87.88 | 94.40 |
|  | *Streptomyces barkulensis* RC 1831 | 87.88 | * | 87.95 |
|  | TRM 70361 | 94.40 | 87.96 | * |
|  | Strains | *Streptomyces carminius* TRM SA0054 | *Streptomyces barkulensis* RC 1831 | TRM 70361 |
| ANIb | *Streptomyces carminius* TRM SA0054 | * | 84.04 | 93.99 |
|  | *Streptomyces barkulensis* RC 1831 | 84.46 | * | 84.55 |
|  | TRM 70361 | 93.65 | 84.23 | * |

Support table 3 Cellular fatty acid contents (per cent) of strain TRM 70351 and its strain TRM 70361 closest relatives.

| fatty acids | TRM 70351 | TRM 70361 |
| --- | --- | --- |
| C_8:0 3OH_ | 4.26% | 6.07% |
| anteiso-C_15:0_ | 15.07% | 5.50% |
| iso-C_16:0_ | 9.53% |  |
| C_16:0_ | 4.69% | 3.65% |
| Sum In Feature 6 | 16.23% | 26.51% |
| 10：0 iso | 2.92% | 3.18% |
| anteiso-C_17:0_ | 11.28% | - |
| Sum Feature | 16.23% | - |
| C_12:0_ | - | 2.44% |
| ISO _14:0_ | - | 2.06% |
| H iso_16：1_ | - | 4.31% |
| iso _16:0_ | - | 9.82% |
| anteiso-C_16:0_ | - | 2.37% |
| Sum In Feature 3 | - | 2.91% |
| anteiso-C_17:0_ | - | 6.48 |

**Note: Only fatty acids with a content greater than 2 per cent are labelled.**

**housekeeping gene**

>TRM 70351 atpD

AGGACACCCATCTCGTCCGCCAGGTTCGGCTGGTAGCCCACCGCGGAGGGCATGCGGCCC

AGCAGCGTGGAGACCTCGGAACCGGCCTGGGTGAAGCGGAAGATGTTGTCGATGAAGAAC

AGCACGTCCTGCTTCTGCACATCGCGGAAGTACTCCGCCATGGTCAGACCGGCCAGCGCC

ACCCGCAGACGGGTGCCCGGGGGCTCGTCCATCTGGCCGAAGACCAGCGCCGTCTGCGGC

AGCACGCCCGACTCGGCCATCTCCTCGATGAGGTCGTTGCCCTCACGGGTGCGCTCACCG

ACACCGGCGAACACGGAGACGCCCTCGTGCAGCTTCGCCACACGCATGATCATTTCCTGG

ATGAGCACGGTCTTGCCGACGCCGGCGCCGCCGAACAGACCGATCTTGCCGCCCTTGACG

TACGGGGTCAGCAGGTCGACGACCTTGAGGCCGGTCTCGAACATCTCGGTCTTGGACTCG

AGCTGGTCGAAG

>TRM 70351 gyrB

TCTGCACGGCGTCGGCGTCTCGGTGGTGAACGCGCTCTCGCAGCGGGTCTCGGTCGACAT

CCGGCGGGACGGTTACCGCTGGACGCAGGAGTACCGGCGCGGCGCGCCCGTCAGTCCGCT

GGAGCGGCAGGAGGCCACCGAGGAGACGGGGACGACGCTCACCTTCTGGGCCGACGGCGA

CATCTTCGAGACGACGGAGTACTCCTTCGAGACGCTGTCGCGGCGTTTCCAGGAGATGGC

GTTCCTCAACGGCGGCCTGACGATCTCGCTGACCGACGAGCGGGCGTCCGCGAAGCAGAC

GGCGGGGGCGGACACCACCGACACGGCGGACGATGACAAGCCCCGTACGGTCACCTACCA

CTACGAGGGCGGGATCTCGGACTTCGTCCGCCATCTCAACTCCCGCAA

>TRM 70351 recA

GACGTCGCCCTCGGCGTCGGCGGCCTGCCCCGGGGCCGGGTCGTCGAGGTGTACGGCCCG

GAGTCCAGCGGCAAGACGACGCTCACGCTGCACGCGGTCGCCAACGCCCAGAAGGCCGGC

GGCACGGTCGCCTTCGTCGACGCCGAGCACGCGCTGGACCCCGAGTACGCCAAGAAGCTC

GGCGTGGACGTCGACCAGCTCATCCTGTCCCAGCCGGACAACGGCGAGCAGGCGCTGGAG

ATCACCGACATGCTCATCAGGTCCGGTGCGCTGGACCTGATCGTGATCGACTCGGTGGCC

GCCCTGGTGCCCCGCGCGGAGATCGAGGGCGAGATGGGCGACTCCCACGTCGGCCTCCAG

GCCCGGCTGATGAGCCAGGCCCTGCGCAAGATCACCGGTGCGCTGCACCAGTCCAGGACG

ACGGCGGTCTTCATCAACCAGCTCCGCGAGAAGGTCGGCGTCATGTTCGGCTCCCCGGAG

ACGACCACCGGCGGCCGGGCGCTG

>TRM 70351 rpoB

GTACGTGGTGTAGGTGCCGTCGTCGTTGGCGACCGTGACGTAGTCGGCGGAGACCTCCTG

GACCACGCCGTCCTTCTCGGCCTTGATGACGTCGCCGGCGTCCACCGCGGAGCGGTACTC

CATGCCGGTGCCGACCAGCGGCGACTCCGCCTTCAGCAGCGGCACCGCCTGGCGCATCAT

GTTCGAGCCCATGAGCGCGCGGTTGGCGTCGTCGTGCTCCAGGAACGGGATCATGGCCGT

GGCCACCGACACCATCTGGCGCGGCGAGACGTCCATGTAGTCGACCTCGTTGCCGGTGAC

GTAGTCGACCTCGCCGCCGCGGCGGCGCACCAGGACGCGGGCCTCGGCGAAGTGCATGTC

GTCGGTCAGCGGCGCGTTGGCCTGCGCGATGACGAAGCGGTCCTCCTCGTCGGCCGTCAG

GTAGTGCGCCTCGTCGGTGACGACGCCGTCCACGACCTTGCGGTACGGCGTCTCGACGAA

GCCGAACGCGTTGACGCGGCCGTAGGAGGCGAGCGAGCCGATCAGGCCGATGTTCGGGCC

>TRM 70351 trpB

CGGCGGAGATCGAGTAGGGCTCGGTGATCTGGCCCTCGTCGTCCTGGAGGACGTAGGAGC

GGGAGCCGTGCAGGATGCCGGGCTCCCCGGCGGTCAGCGTCGCCGCGTGCTCGCCGGAGT

CCACGCCGTGGCCGGCGGCCTCCAGCCCGACCAGCCGCACGGCGGCGTCGCCGAGGAACG

CGTGGAAGAGGCCGATGGCGTTGGAGCCGCCGCCCACGCAGGCGGCGACGGCGTCCGGCA

GCCGCCCGGTGCGCTCCAGGATCTGGCGGCGGGCCTCGACGCCGATCACCCGGTGGAAGT

CGCGGACGAGGGCGGGGAACGGGTGCGGTCCGGCGACGGTGCCGAACAGGTAGTGGGTGT

CCTCCACGTTGGCGACCCAGTCGCGGAACGCCTCGTTGATGGCGTCCTTGAGGGTGCGGC

TGCCGGAGGCGACGGGGACGACCTCGGCGCCCAGCATGCGCATCCGGGCGACGTTGAGCG

CCTGGCGCCGGGTGTCGATCTCGCCCATGTAGATCGTGCAGTCGAGGCCGAACAGGGCGC

AGGCGGTGGCGGTGGCCACGCCGTGCTGCC

>TRM70361atpD

CCCATCTCGTCCGCCAGGTTCGGCTGGTAGCCCACCGCGGAGGGCATGCGGCCCAGCAGG

GTGGAGACCTCGGAACCGGCCTGGGTGAAGCGGAAGATGTTGTCGATGAAGAACAGCACG

TCCTGCTTCTGGACGTCGCGGAAGTACTCCGCCATCGTCAGACCGGCCAGGGCGACCCTC

AGCCGGGTGCCCGGCGGCTCGTCCATCTGCCCGAAGACCAGCGCGGTCTGCGGGAGAACG

CCGGACTCCTCCATCTCGACGATGAGGTCGTTGCCCTCACGGGTGCGCTCGCCGACGTCG

GCGAACACGGAAACGCCCTCGTGCAGCTTCGCCACACGCATGATCATTTCCTGGATGAGG

ACGGTCTTGCCGACGCCCGCGCCGCCGAACAGACCGATCTTGCCGCCCTTGACGTACGGG

GTCAGCAGGTCGATGACCTTCAGACCGGTCTCGAACATCTCGGTCTTGGACTCGAGCTGG

TCGAAGTTGCGGGAGTTGAGGTGGCGGACGAAGTCCGAGATGCCGCCCTCGTAGTGGTAGCGCACG

>TRM70361gyrB

AAACGCGGCTTGTCGTCCTCCGCCGTGTCCGTGGTGTCGGCGCCCGCCGTCTGCTTGGCC

GCCTCCCGCTCGTCGGTCAGAGTGATCGTCAGACCGCGGTTGAGGAACGCCATCTCCTGG

AAGCGCCGCGAGAGCGTCTCGAAGGAGTAGTCGGTCGTGTCGAAGATGTCGCCGTCGGCC

CAGAAGGTGACCGTCGTACCGGTCTCGTCGGTGGCCTCGTTGCGCTGGAGGGGCGCGGTG

GGCGCGCCCATCTTGTACTCCTGCGTCCAGCGGTAGCCGTCCCGCTTGACCTCGACCGAG

ACCTTGGTCGACAGCGCGTTGACCACCGAGACGCCCACCCCGTGCAGAGACGTGGCCCTCGGGGTGGGCGGTCTGCCGCGCGGCCGGGTGGTCGAGGTCTACGGCCCG

>TRM 70361 recA

GAGTCCTCCGGCAAGACGACCCTGACCCTCCACGCGGTGGCCAACGCCCAGCGGATGGGC

GGCACCGTCGCCTTCGTGGACGCCGAGCACGCGCTGGACCCGGAGTACGCCAAGCGGCTG

GGTGTGGACGTGGACTCCCTCATCCTGTCCCAGCCGGACAACGGCGAGCAGGCGCTGGAG

ATCACCGACATGCTGATCCGCTCCGGCGCCCTCGACCTGATCGTGATCGACTCCGTGGCC

GCCCTGGTGCCGCGCGCGGAGATCGAGGGCGAGATGGGCGACTCCCACGTCGGCCTCCAG

GCCCGGCTGATGAGCCAGGCGCTGCGGAAGATCACCGGTGCGCTCCACCAGTCCAAGACC

ACCGCGATCTTCATCAACCAGCTCCGCGAGAAGGTCGGCGTGATGTTCGGCTCCCCGGAG

ACCACCACCGGTGGCCGGGCGCTGGGGCCGAACATCGGTCTGATCGGTTCGCTGGCCTCGTACGGCCGGGTGAACGCGTTCGGC

>TRM 70361 rpoB

TTCGTCGAGACCCCGTACCGCAAGGTCATCGACGGCCAGGTCACCGACGAGGTGAACTAC

CTGACCGCCGACGAGGAGGACCGCTACGTCATCGCGCAGGCCAACGCGCCGCTGACGGAC

GACATGCGGTTCGCCGAGGGCCGGGTCCTGGTCCGCCGCCGCGGCGGCGAGGTCGACTAC

GTGCCCGGCGGCGAGGTCGACTACATGGACGTCTCGCCGCGCCAGATGGTGTCGGTCGCG

ACCGCCATGATCCCGTTCCTGGAGCACGACGACGCCAACCGCGCGCTCATGGGCTCGAAC

ATGATGCGCCAGGCCGTCCCGCTGATCCGGGCCGAGTCCCCGCTGGTCGGCACCGGCATG

GAGTACCGCTCCGCGGTCGACGCCGGTGACGTGATCAAGGCCGAGAAGGACGGCGTGGTC

CAGGAGGTCTCCGCCGACTACGTCACGGTGGCCAACGACGACGCCACGTACACCACGTACCGGCCGAGATGGAGTACGGCTCGGTGATCTGGCCCTCGTCGTCCTGGAGTACGTAGGAGC

>TRM 70361 trpB

GCGAGCCGTGCAGGATGCCCGGCTCCCCGGCGGTCAGCGTCGCCGCGTGCTCGCCGGAGG

CCACCCCGTGGCCGGCCGCCTCCAGGCCCACCAGCCGGACGCCGGTGTCGGGGATGAAGG

CGTGGAACAGGCCGATCGCGTTGGAGCCGCCGCCGACGCAGGCCAGCACCGCGTCCGGCA

GCCGTCCGGTGCGCTCCAGGATCTGCCGCCGGGCCTCCACACCGATGACCCGGTGGAAGT

CGCGCACCAGCGCCGGGAAGGGGTGGGGTCCGGCGACGGTGCCGAAGAGGTAGTGGGTGT

GGTCGACGTTGGCGACCCAGTCGCGGAACGCCTCGTTGATGGCGTCCTTCAGGGTCCGGC

TGCCGGAGGTCACCGGGATCACCTCGGCGCCCAGCATCCGCATCCGGGCGACGTTGAGCG

CCTGGCGCTGGGTGTCGATCTCGCCCATGTAGACGGTGCACTCCAGGCCGAACAGGGCGC

AGGCGGTGGCCGTGGCGACGCCGTGCT
